# Diversity of organotrophic bacteria, activity of dehydrogenases and urease as well as seed germination and root growth *Lepidium sativum*, *Sorghum saccharatum* and *Sinapis alba* under the influence of polycyclic aromatic hydrocarbons

**DOI:** 10.1007/s11356-015-5329-2

**Published:** 2015-09-05

**Authors:** Aneta Lipińska, Jadwiga Wyszkowska, Jan Kucharski

**Affiliations:** Department of Microbiology, University of Warmia and Mazury in Olsztyn, Plac Lodzki 3, 10-727 Olsztyn, Poland

**Keywords:** Polycyclic aromatic hydrocarbons, Colony development index, Ecophysiological diversity index, Soil resistance, Phytotoxkit test

## Abstract

Polycyclic aromatic hydrocarbons are organic compounds with highly toxic, carcinogenic, and mutagenic properties, which adversely affect the basic biological parameters of the soil, including the count of microorganisms, and the enzymatic activity. In addition to disturbances to the biological activity of the soil, PAHs may also exhibit toxic effects on plants. In view of the above, the study involved testing aimed at the determination of the effects of polycyclic aromatic hydrocarbons in a form of naphthalene, phenanthrene, anthracene and pyrene on the count, colony development (CD) index, ecophysiological (EP) diversity index of organotrophic bacteria, and the activity of soil dehydrogenases and soil urease. Moreover, an attempt was made to determine the soil’s resistance based on the activity of the above-listed enzymes, and the effect of polycyclic aromatic hydrocarbons on seed germination and root growth was assessed by *Lepidium sativum*, *Sorghum saccharatum*, and *Sinapis alba*. In addition, the species of bacteria found in a soil subjected to strong pressure of polycyclic aromatic hydrocarbons were isolated. The experiment was performed in a laboratory on samples of loamy sand. Polycyclic aromatic hydrocarbons were introduced into the soil in an amount of 0, 1000, 2000, and 4000 mg kg^−1^ of soil dry matter. Germination and growth of cress (*L. sativum*), white mustard (*S. alba*), and sweet sorghum (*S. saccharatum*) were determined using Phytotoxkit tests. It was found that the tested PAHs increased the average colony counts of organotrophic soil bacteria; pyrene did so to the greatest extent (2.2-fold relative to non-contaminated soil), phenanthrene to the smallest extent (1.4-fold relative to non-contaminated soil). None of the PAHs changed the value of the bacterial colony development (CD) index, while anthracene and pyrene increased the value of the eco-physiological (EP) diversity indicator. PAHs lowered the activity of the tested enzymes. The activity of dehydrogenases was dependent on a greater extent by the type of hydrocarbon (54.56 %) rather than by the dose (10.64 %), while for the activity of urease, it was the opposite. The greater extent was dependent on dose (95.42 %) rather than by type (0.21 %). Dehydrogenases are characterised by greater resistance to the action of PAHs than urease. Based on seed germination and root growth, it has shown that *S. alba* is best suited, being the most vulnerable plant, while *S. saccharatum* is the least suited. Subjecting a soil to strong pressure of PAHs leads to disturbances to the biological parameters of the soil, seed germination, and root growth *L. sativum*, *S. saccharatum*, and *S. alba*.

## Introduction

Environmental pollution with polycyclic aromatic hydrocarbons (PAHs) from oil spills, industrial processes, and atmospheric deposition poses a serious threat for water and land ecosystems (Driscoll et al. 2010; Maliszewska-Kordybach et al. 2013). Ecological functions, food safety, and human health are determined by the pool of PAHs released into the environment (Li et al. 2013). Soil is a major reservoir for PAHs. Those toxic, carcinogenic, and lipophilic compounds (Amezcua-Allieri et al. 2012) bind to soil organic matter, which contributes to their persistence and stability in the soil environment (Wyszkowski and Ziółkowska 2013). PAHs significantly influence soil biological parameters (microbial counts and enzyme activity) that are the key determinants of soil quality. The results of various studies indicate that the stimulating or inhibitory effect of PAHs on microbial abundance and enzyme activity cannot be unequivocally established. Polycyclic aromatic hydrocarbons alter the microbiological and biochemical properties of soils (Wyszkowska and Kucharski 2000; Shen et al. 2005a, b). In addition, the action of PAHs is better represented by the soil enzymes, in particular dehydrogenases and urease, than the count of microorganisms (Wyszkowska et al. 2008; Lipińska et al. 2014a).

PAHs disrupt the biological activity of soil, and they can also exert toxic effects on plants, the basic source of raw materials for production. The inhibition of physiological processes in plants, which is manifested by chlorosis, yellowing, and reduced growth parameters, is one of the most characteristic symptoms of PAH contamination (Meudec et al. 2007). Therefore, our study tested the effects of naphthalene, phenanthrene, anthracene, and pyrene on seed germination and root growth *Lepidium sativum*, *Sorghum saccharatum*, and *Sinapis alba*.

Soil pollution with PAHs can be reversed through the application of remediation techniques that eliminate contaminating compounds and restore natural soil conditions. Soil remediation methods are expensive, which justify the continued search for cheaper and environmentally-friendly techniques. Bioremediation (Lin et al. 2010) is an effective method that relies on microorganisms capable of degrading PAHs. Microbial growth rates and, consequently, the rate and quality of biodegradation are determined by several factors, including temperature, soil moisture content, pH, microbial diversity, and nutrient availability (Yessicaa et al. 2013). PAHs can be degraded by bacteria, fungi (Zafra et al. 2015), yeasts, and algae (Bundy et al. 2004), but bacteria play the key role in the process. Due to the natural potential of microorganisms that possess enzymatic capabilities, bioremediation is more effective when microbial consortia are used instead of individual isolates. Numerous bacterial genera (*Pseudmonas*, *Bacillus*, *Micrococcus*, *Mycobacterium*, *Vibrio*, *Paenibacillus*, *Corynebacterium*, *Staphylococcus*, and *Aeromonas*) have been identified and characterized based on their ability to degrade PAHs (Lin et al. 2010; Pedetta et al. 2013). The first stage in microbiological degradation of PAHs involves dioxygenase that inserts oxygen atoms between two carbon atoms in the benzene ring of a given PAH. The resulting cis-dihydrodiol is rearomatized by dehydrogenases to produce indirect dihydroxylyzed forms that cleave the ring and generate Krebs cycle products (Kanaly and Harayama 2000). Despite the wide availability of bacteria capable of metabolizing PAHs, new species are being discovered and subjected to genetic enhancement. For instance, the presence of the catA gene (catechol 1,2-dioxygenase gene) and the alkB gene (alkane 1-monooxygenase) indicates that selected species such as *Gordonia* spp. can be potentially used for bioremediation (Shen et al. 2009, 2010).

In view of the above, the study involved testing aimed at the determination of the effects of polycyclic aromatic hydrocarbons in a form of naphthalene, phenanthrene, anthracene, and pyrene on the count, colony development (CD) index, ecophysiological (EP) diversity index of organotrophic bacteria, and the activity of soil dehydrogenases and soil urease. Moreover, an attempt was made to determine the soil’s resistance based on the activity of the above-listed enzymes, and the effect of polycyclic aromatic hydrocarbons on seed germination and root growth was assessed by *L. sativum*, *S. saccharatum*, and *S. alba*. In addition, the species of bacteria found in a soil subjected to strong pressure of polycyclic aromatic hydrocarbons were isolated.

## Materials and methods

### Soil

The experimental material comprised surface soil samples collected from the uppermost soil horizon (0–20 cm) at the Educational and Experimental Center in Tomaszkowo (NE Poland). The soils were classified as Eutric Cambisol on the World Reference Base of Soil Resources (2014). According to the graining classification of the United States Department of Agriculture, it was a soil with a granulometric composition of loamy sand (sand fraction—72.42 %, silt fraction—25.31 %, and colloidal clay fraction—2.27 %). The samples were dried and passed through a 2-mm mesh sieve. Grain-size composition of the soil was determined with laser method using a Mastersizer 2000. The pH of soil in 1 mol KCl dm^−3^ was determined at 7.15, hydrolytic acidity at 4.8 mmol(+) kg^−1^, total exchangeable base cations at 279 mmol(+) kg^−1^, organic carbon content at 13.1 g kg^1^, total nitrogen content at 1.1 g kg^−1^, available phosphorus content at 200 mg kg^−1^, potassium content at 147 mg kg^−1^, and magnesium content at 27 mg kg^−1^. Soil pH was determined by potentiometry in aqueous KCl solution of the concentration of 1 mol KCl dm^−3^, hydrolytic acidity, and total exchangeable bases by the Kappen’s method (Carter 1993), content of organic carbon—with Tiurin’s method (Nelson and Sommers 1996), content of total nitrogen according to the method by Kjeldahl (ISO 11261. 1995), available phosphorus, potassium, and magnesium content by methods exactly described in the publication Kucharski and Wyszkowska (2000). The physicochemical properties of the soil were determined in three replications.

### Polycyclic aromatic hydrocarbons

Four organic compounds, naphthalene (B&K Multi-branch Company), phenanthrene, anthracene, and pyrene (Acros Organics), were used as the model PAHs in the experiment. All of the tested compounds have been designated as priority pollutants by the US Environmental Protection Agency (US EPA). Soil samples were contaminated with powdered PAHs in the amount of 0, 1000, 2000, and 4000 mg kg^−1^ dry matter of soil. The application of such high doses was justified by the amounts of PAHs that are released into the soil environment as a result of frequent and uncontrolled spills of petroleum derivatives (Park and Park 2011). Naphthalene, phenanthrene, anthracene, and pyrene are characterized by n-octanol/water partition coefficients of 3.6, 4.46, 4.5, and 4.8, respectively; they are insoluble in water, but soluble in organic compounds.

### Experimental design

One hundred fifty cubic centimeter glass beakers were filled with 100 g of soil. Ammonium nitrate (NH_4_NO_3_) was added to each soil sample in the amount of 100 mg kg^−1^ dry matter of soil. The samples were contaminated with PAHs in the described doses. The samples were mixed, and demineralized water was added to 50 % capillary water capacity. The samples were incubated for 32 weeks at room temperature without light access. Soil moisture was kept constant during incubation through the addition of demineralized water. The counts and structure of organotrophic bacteria and the activity of dehydrogenases and urease were determined after incubation. Microorganisms were isolated from soil samples and identified to the species level by BLIRT (BioLab Innovative Research Technologies) of Gdańsk.

### Determination of organotrophic bacteria counts

Specimens of 10 g were collected from every soil sample and suspended in sterile saline solution to determine the population size of organotrophic bacteria. A series of dilutions were performed, and 1 cm^3^ of the selected suspension was plated in Bunt and Rovira medium (Bunt and Rovira 1955). The counts of organotrophic bacteria were determined in three replications. Organotrophic bacteria were counted daily for 10 days with the use of a colony counter, and microbial counts were determined based on the below formula:

$$ \mathrm{cfu}=\frac{\mathrm{a}\cdot \mathrm{n}\cdot 100}{\%\mathrm{D}\mathrm{M}}\cdot {10}^3 $$

where a is the number of colonies in a plate; n is the inverse of the dilution factor; 100 %^−1^ DM is the conversion factor to dry matter basis; 10^3^ is the conversion factor per 1 kg of soil.

### Determination of dehydrogenase and urease activity

Dehydrogenase and urease activity was determined in three replications according to the methods proposed by Öhlinger (1996) and Alef and Nannipieri (1998). The substrate for dehydrogenase was 3 % triphenyl-tetrazolium chloride (TTC) solution and for urease–urea. Soil samples were incubated for 24 h at 37 °C, and extinction was measured with the Aquarius CE7500 spectrophotometer (Cecil Instruments) at 485 nm for dehydrogenases and 410 nm for urease. The results were expressed in micromole TPF (triphenyl formazan) per kilogram soil DM per hour for dehydrogenases and in millimole N-NH_4_ per kilogram soil DM per hour for urease.

### Determination of indicators of colony development, ecophysiological diversity, and soil resistance values

Indicators of colony development (CD) and ecophysiological diversity (EP) were calculated to determine changes in microbial diversity of PAH-contaminated soil after 32 weeks of incubation. CD values were calculated based on the formula proposed by Saratchandra et al. (1997):

$$ \mathrm{C}\mathrm{D}=\left({\mathrm{N}}_1/1+{\mathrm{N}}_2/2+{\mathrm{N}}_3/3+\dots +{\mathrm{N}}_{10}/10\right)\cdot 100 $$

where CD is the colony development index, N_1_, N_2_, N_3_, …, N_10_ are the number of microbial colonies on day 1, 2, 3, …, 10, expressed as a percentage of the total number of organotrophic bacteria counted daily for 10 days. The higher the value of CD, the higher the rate of bacterial proliferation in the course of one or several days.

EP values were calculated using the formula proposed by DeLeij et al. (1994):

$$ \mathrm{E}\mathrm{P}=-\sum \left({\mathrm{p}}_{\mathrm{i}}\cdot \log 10{\mathrm{p}}_{\mathrm{i}}\right) $$

where EP is the ecophysiological diversity index, p_i_ is the number of organotrophic bacterial colonies on a given day divided by the total number of microbial colonies. The higher the value of ED, the more uniform the proliferation of microbial colonies in the analyzed period.

Soil resistance (RS) to contamination with PAHs was determined based on the formula proposed by Orwin and Wardle (2004):

$$ \mathrm{R}\mathrm{S}\left({\mathrm{t}}_0\right)=1-\frac{2\left|{\mathrm{D}}_0\right|}{\left({\mathrm{C}}_0+\left|{\mathrm{D}}_0\right|\right)} $$

where RS is the soil resistance index, D_0_ is the difference between control soil (C_0_) and PAH-contaminated soil after 32 weeks of incubation (t_0_). The values of RS range from of −1 to 1, where −1 and 0 imply that the pollutant has a very strong impact (200 and 100 %, respectively) on soil. The closer the RS values are to 1, the higher the soil’s resistance to contamination, indicating that PAHs have a negligent effect or no effect on soil.

### Effect of PAHs on seed germination and root growth of *Lepidium sativum*, *Sinapis alba*, and *Sorghum saccharatum*

Effect of PAHs on germination and growth of cress (*L. sativum*), white mustard (*S. alba*) and sweet sorghum (*S. saccharatum*) were determined using Phytotoxkit tests. After 32 weeks of incubation, 110 g samples of control soil and soil contaminated with a PAH dose of 4000 mg kg^−1^ dry matter of soil were placed on plastic plates. Ten seeds of each of the tested plants were placed on filter paper in three replications. The plates were incubated in vertical orientation at 25 °C for 3 days. After incubation, percent inhibition of seed germination (SG) and percent inhibition of root growth (RI) were determined with the use of the below formula:

$$ \mathrm{S}\mathrm{G}=\left(\mathrm{A}-\mathrm{B}/\mathrm{A}\right)\cdot 100,\mathrm{R}\mathrm{I}=\left(\mathrm{A}-\mathrm{B}/\mathrm{A}\right)\cdot 100 $$

where A is the seed germination and root growth in control samples; B is the seed germination and root growth in experimental samples.

### Characterization of organotrophic bacteria

Before culturing, 1 g specimens were collected from each soil sample (one control sample and four soil samples contaminated with naphthalene, phenanthrene, anthracene, and pyrene doses of 4000 mg kg^−1^ dry matter of soil and suspended in sterile saline solution at 1:10. After serial dilutions with saline solution, 1 cm^3^ of the resulting solution was placed on culture media in three replications.

In order to check the number of different groups of microorganisms, the following media were used for the isolation:

- PCA for determining total bacterial counts in food products, water, soil, and other substrates; cultures were prepared by the pour plate method, and the plates were incubated at 37 °C for 96 h;
- Baird-Parker selective agar for determining coagulase-positive staphylococci. The applied medium contains lithium chloride that inhibits the growth of associated microorganisms; cultures were prepared by the spread plate method, and the plates were incubated at 37 °C for 48 h;
- Sabouraud medium with chloramphenicol for determining molds, where the antibiotic inhibits the growth of nearly all associated microorganisms; cultures were prepared by the pour plate method, and the plates were incubated at 30 °C for 96 h;
- VRBG selective agar for determining microorganisms of the family *Enterobacteriaceae*; due to the inclusion of the natural red indicator, bacteria that metabolize glucose into acids produce pink to red colonies that are often surrounded by a band of strontium yellow; cultures were prepared by the pour plate method, and the plates were incubated at 37 °C for 48 h.

After the determination of total microbial counts in PCA, the most characteristic and most rapidly proliferating colony was selected from each of the five Petri plates (one control sample and four experimental samples contaminated with PAHs). The selected colonies were labeled as K_PCA_, N_PCA_, F_PCA_, A_PCA_, and P_PCA_. Every colony was transferred to an separate probe containing liquid PCA. The same procedure was applied to sample colonies from Baird-Parker agar, and the selected colonies were labeled as K_BP_, N_BP_, F_BP_, A_BP_, and P_BP_. All cultures were incubated at 37 °C for 24 h.

Genomic DNA was extracted from strains with the use of the EXTRACTME DNA BACTERIA KIT (DNA Gdańsk). Products in the 16S–23S rDNA intergenic spacer region were amplified using PCR reagents supplied by DNA Gdańsk (Poland). Forward (AGA GTT TGA TCC TGG CTC AG) and reverse (GTG TGA CGG GCG GTG TGT AC) primers generating PCR products with the size of 1400 bp were used in amplification. For the PCR reaction, a reaction mixture was used with the following composition: 2.5 mm^3^ of buffer, 5 mm^3^ of Q-solution, 1.6 mm^3^ of a solution of four deoxyribonucleoside triphosphates (dNTP), 1 mm^3^ of MgCl_2_ (magnesium ions are cofactors of *Taq* polymerase), a couple of starters (reverse and forward), 7.65 mm^3^ of water free of RNases, and 0.25 mm^3^ of *Taq* polymerase. The conditions of the PCR reaction were the following: initial denaturation temperature—95 °C, time—3 min; denaturation temperature—95 °C, time—0.5 min; heating temperature—57 °C, time—1 min; elongation temperature—72 °C, time—1 min; final elongation temperature—72 °C, time—7 min; cooling temperature—4 °C; number of cycles—35.

After the PCR reaction, reaction mixtures were separated in 1.5 % agarose gel to inspect the quality of amplified products. PCR products were extracted from gel and purified with the use of the EXTRACTME DNA GEL-OUT KIT. PCR products were sequenced in both directions in the 3730 XL DNA Analyzer (Life Technologies). The results were analyzed with the use of the NCBI database and BLAST software.

In the group of the examined bacterial strains, nucleotide sequences with the length of 1400 bp were selected based on their percentage share in the identified species. All sequences were aligned in Clustal W (MEGA 6.0 tool). The phylogenetic tree was developed by the neighbor-joining method.

### Statistical analysis

The results of laboratory tests performed in three replications were processed by ANOVA in the Statistica 9.1 application (StatSoft 2010). The homogeneity of variance was analyzed by Tukey’s test at a significance level of *p* = 0.01. Dehydrogenase and urease activity and the counts of organotrophic bacteria were visualized by PCA. The proportion of variation attributed to dehydrogenase and urease activity and the counts of organotrophic bacteria was determined by eta-squared calculations. The coefficients of correlation between the degree of soil contamination with PAHs and the abundance of organotrophic bacteria, dehydrogenase and urease activity, and soil resistance were determined in Excel.

## Results and discussion

### Abundance and structure of organotrophic bacteria in PAH-contaminated soil

**Fig. 1:**
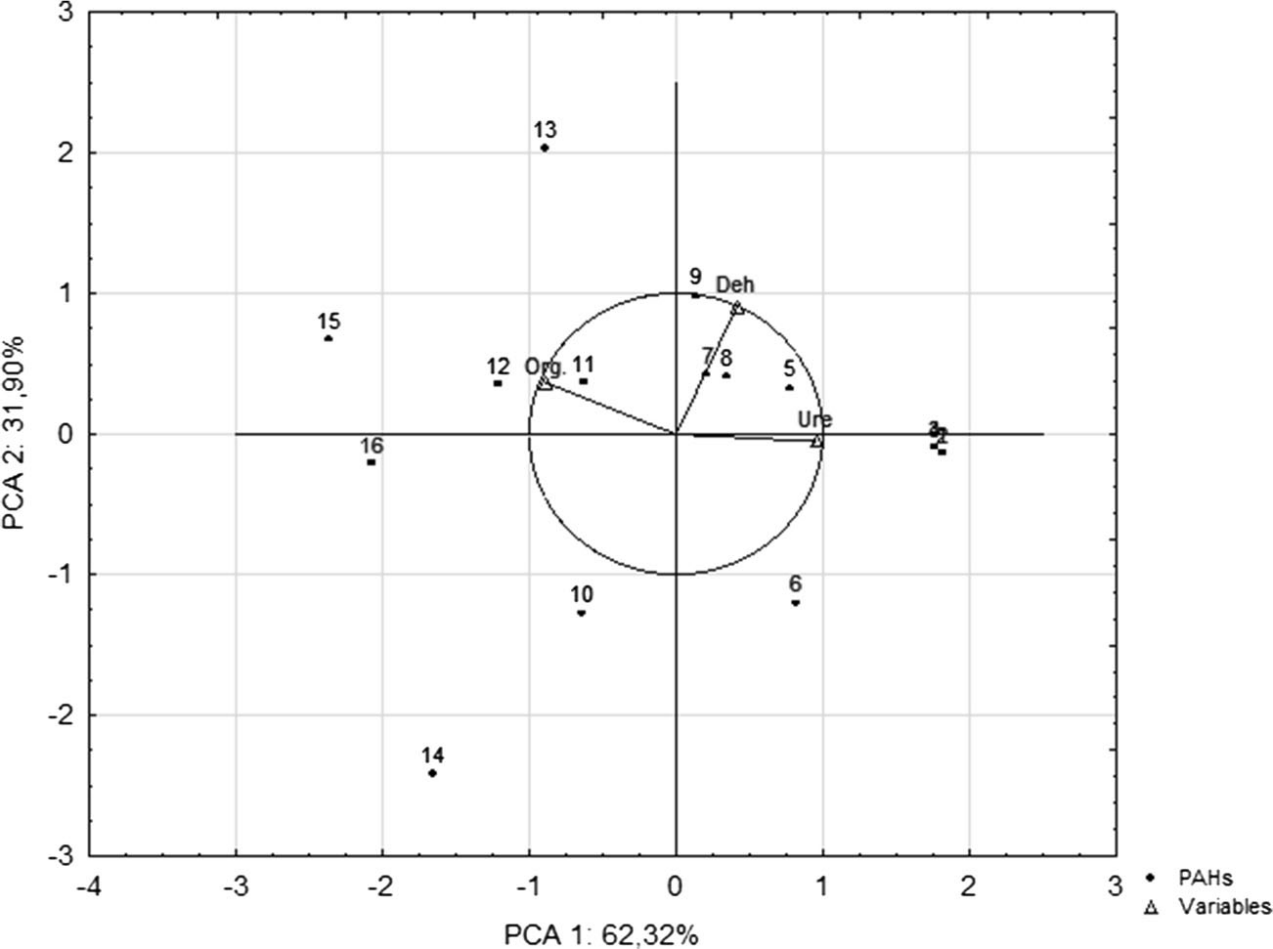
The number of organotrophic bacteria, dehydrogenase, and urease activity in loamy sand contaminated with PAHs determined by the PCA method. Explanations: Org.—organotrophic bacteria, Deh—dehydrogenases; Ure—urease; 1–4—control samples without PAHs, 5–8—samples contaminated with naphthalene, phenanthrene, anthracene, and pyrene doses of 1000 mg kg^−1^ soil DM, respectively; 9–12—samples contaminated with naphthalene, phenanthrene, anthracene, and pyrene doses of 2000 mg kg^−1^ soil DM, respectively; 13–16—samples contaminated with naphthalene, phenanthrene, anthracene, and pyrene doses of 4000 mg kg^−1^ soil DM, respectively

High doses of PAHs initially inhibit microbial growth when soil-dwelling microorganisms come into contact with new environmental stressors. After a period of stagnation in soils contaminated with PAHs, new populations of bacteria, Actinobacteria and fungi emerge, and microbial metabolism lead to the degradation of PAHs (Baran et al. 2004; Shen et al. 2005a). The counts of organotrophic bacteria varied under the influence of PAHs and were significantly correlated with the dose and type of the applied PAHs, which explained variation in 63.59 and 17.35 %, respectively (Table 1). The abundance of the analyzed bacteria increased with a rise in contamination levels, and the highest average colony counts were noted in soil samples treated with pyrene (Table 2). The pyrene dose of 4000 mg kg^−1^ dry matter of soil increased the abundance of organotrophic bacteria 2.9-fold relative to control. Pyrene, as the first of the PAHs, is included in the group of hydrocarbons of high molecular weight, which in turn leads to its increased stability in the soil, and translates into a stronger effect towards microorganisms. Even after a period of 32 weeks, microorganisms were able to use this hydrocarbon as a carbon donor. The variable representing organotrophic bacterial counts in the PCA diagram (Fig. 1) lies closer to the horizontal axis and explains 62.32 % of the variation. The lowest average microbial abundance was observed in soil samples contaminated with phenanthrene, which is illustrated by the greatest distance between points 6, 10, and 14 and the vector representing organotrophic bacteria. In initial stages, PAHs constitute a robust source of energy and carbon for microorganisms whose energy demand increases under exposure to new environmental stressors (Margesin et al. 2000). Wyszkowska and Kucharski (2005) reported on Diesel oil’s stimulating effect on the counts of bacteria, *Actinobacteria*, copiotrophic bacteria, spore-forming copiotrophic bacteria, and oligotrophic bacteria. The development of organotrophic bacteria could be determined by the introduction of high doses of PAHs to the soil, but having considered the decreasing activity of dehydrogenases, a different reason for the increasing count of microorganisms may be assumed. Following the application of naphthalene, phenanthrene, anthracene, and pyrene to the soil, some microorganisms could probably have died in the soil. This is why a lowered activity of dehydrogenases was recorded in the study. The cells of microorganisms which were lysed provided a substrate to other microorganisms occurring in the soil. Hence, the increased count of bacteria was found (Hicks et al. 1990).

**Table 1:** Percentage of independent variables factors (type of PAHs and dose of PAHs) η^2^ in the formation number of organotrophic bacteria and soil dehydrogenases and urease activity

Variables	Organotrophic bacteria	Dehydrogenases	Urease
Type of PAHs	17.35	54.56	0.21
Dose of PAHs	63.59	10.64	95.42
Type dose of PAHs	13.86	33.66	3.40
Error	5.20	1.14	0.96

η^2^ processed by ANOVA in the Statistica 9.1.

**Table 2:** The number of organotrophic bacteria in soil contaminated with PAHs

Dose of PAHs (mg kg^−1^ DM soil)	Type of PAHs			
	Naphthalene	Phenanthrene	Anthracene	Pyrene
	10^9^ cfu kg^−1^ DM soil			
0	18.25 a^a^	18.25 a	18.25 a	18.25 a
1000	23.25 b	22.47 b	32.25 b	35.72 b
2000	34.22 c	27.67 bc	39.48 c	50.68 c
4000	44.36 d	31.12 c	63.70 d	52.79 c
average	30.02	24.87	38.42	39.36
*r*	0.98	0.96	0.99	0.88

^a^In columns homogenous groups are followed by the same letter

*r* correlation coefficient

**Fig. 2:**
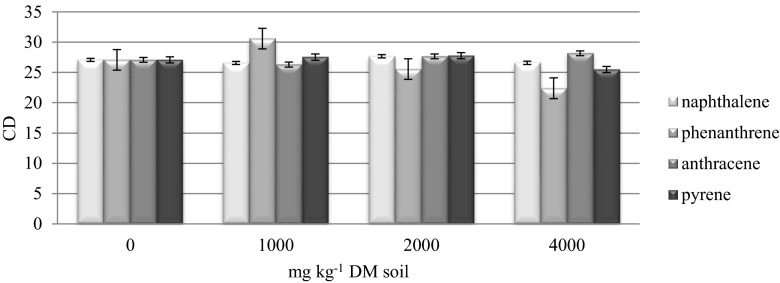
Indices of colony development (CD) of organotrophic bacteria in soil contaminated with PAHs. Error bars represent standard deviation (*n* = 9)

According to the r/K selection theory, genetic differences between microorganisms enable microbes to survive in diverse environments (De Leij et al. 1994). The development of bacterial colonies on each day of the analyzed period can be monitored with the use of CD index values in the range of 0 to 100. CD values are higher when microbial populations proliferate within a short period of time relative to the entire experimental period. In this study, CD values remained fairly stable regardless of the dose and type of PAHs (Fig. 2). The highest average value of CD was noted in samples treated with anthracene (27.27) and the lowest in samples contaminated with phenanthrene (26.38). Our findings could indicate that microorganisms had successfully adapted to an environment contaminated with PAHs. After 32 weeks of incubation, there was a high probability of development towards K-strategists and slow-growing microorganisms that easily adapt to changing environmental conditions (DeLeij et al. 1994; Saratchandra et al. 1997), even after a period of significant contamination with PAHs. The above assumption is validated by the identification of bacterial species, most of which were autochthonous microbes (K-strategists) with possible genetic changes that contribute to survival in unsupportive environments.

**Fig. 3:**
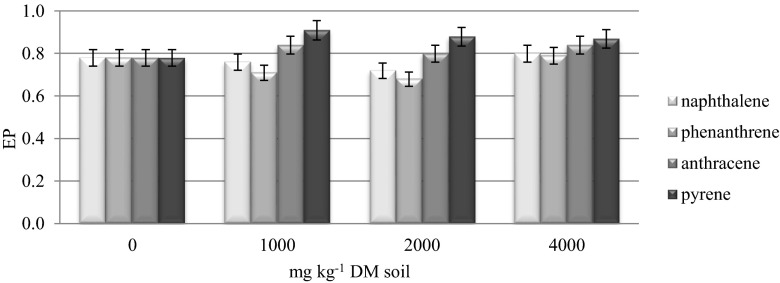
Ecophysiological diversity index (EP) of organotrophic bacteria in soil contaminated with PAHs. Error bars represent standard deviation (*n* = 9)

The CD index is related to the EP index whose values ranged from 0.68 to 0.91, subject to the applied dose and type of PAHs. The highest average EP values were reported in soil treated with pyrene (Fig. 3) (0.86), and the lowest EP values were reported in samples contaminated with phenanthrene (0.74). Bacterial proliferation could be conditioned by the bioavailability of PAHs resulting from the structure of those organic compounds. EP values reflect the uniformity of growth of orghanotrophic bacteria over a given period of time. EP values range from 0 to 1, and the higher the value of the indicator, the more uniform the growth of the examined microorganisms.

### Dehydrogenase and urease activity in PAH-contaminated soil

The quantity and quality of pollutants introduced to the soil environment significantly affect enzymatic activity, which is one of the key biological indicators of soil quality. Dehydrogenases and urease are enzymes that are most sensitive to external stressors, and in contaminated substrates, their activity is stimulated or inhibited relative to control. In this study, the PAH dose significantly influenced dehydrogenase and urease activity. The PCA diagram (Fig. 1) presents the activity levels of dehydrogenases and urease and points representing different doses of PAHs. The urease activity vector lies closer to the horizontal axis, and it explains 62.32 % of the variation (Fig. 1). Urease activity was highly significantly correlated with the PAH dose, and it explained 95.42 % of the variation (Table 1). Urease activity decreased with an increase in the PAH dose (Table 4), and the observed changes were significant already at the dose of 1000 mg kg^−1^ dry matter of soil, which is reflected by the proximity of points 5–8 representing the above dose in the PCA chart. Variations in dehydrogenase activity were explained by the PAH dose in 10.64 % (Table 1) and by the type of the applied PAH in 54.56 %. Dehydrogenase was most strongly inhibited by phenanthrene, which is demonstrated by the greatest distance between points 6, 10, and 14 (Fig. 1) and the vectors of variables. Anthracene had the least inhibitory effect on dehydrogenase activity (Table 3), whereas urease activity was least suppressed by pyrene (Table 4). Shen et al. (2005a, b) demonstrated that phenanthrene, fluoranthene, and benzo(a)pyrene had a negative effect on the activity of the discussed enzymes. PAHs are highly toxic, and they inhibit most soil enzymes (Wyszkowska and Kucharski 2000; Lipińska et al. 2013). In some reports, however, PAHs had a stimulating effect on the biochemical activity of soil. Klimkowicz-Pawlas and Maliszewska-Kordybach (2003) evaluated the impact of anthracene and pyrene on dehydrogenase activity and reported an increase in enzymatic activity in samples freshly contaminated with PAHs. In a study of soil contaminated with diesel oil doses of 3, 6, 9, 12, 24, and 10 cm^3^ kg^−1^ dry matter of soil, the activity of dehydrogenases, urease, and alkaline phosphatase increased relative to control (Wyszkowska et al. 2006; Wyszkowska et al. 2008). The number of benzene rings determines a PAH’s ability to stimulate enzymatic activity. Organic compounds containing three or four rings constitute a rich source of energy and carbon for microorganisms, whereas compounds containing a higher number of rings are toxic, mutagenic, and carcinogenic (Baran et al. 2004; Shen et al. 2005b). Regardless of the applied PAH dose, the lowest average level of dehydrogenase activity was noted in soil treated with phenanthrene. The above results could be linked to the counts of organotrophic bacteria, which were also lowest in soil contaminated with phenanthrene. Even high doses of the analyzed pollutant could have been utilized in early stages of the experiment.

**Table 3:** Activity of dehydrogenases in soil contaminated with PAHs

Dose of PAHs (mg kg^−1^ DM soil)	Type of PAHs			
	Naphthalene	Phenanthrene	Anthracene	Pyrene
	μmol TFF kg^−1^ DM soil h^−1^			
0	8.62 c^a^	8.62 c	8.63 b	8.63 c
1000	8.93 c	7.10 b	8.73 b	8.62 c
2000	7.25 b	6.73 b	8.37 ab	7.98 b
4000	6.21 a	5.15 a	7.87 a	7.16 a
average	7.75	6.90	8.40	8.10
*r*	−0.93	−0.98	−0.94	−0.97

^a^In columns homogenous groups are followed by the same letter

*r* correlation coefficient

**Table 4:** Activity of urease in soil contaminated with PAHs

Dose of PAHs (mg kg^−1^ DM soil)	Type of PAHs			
	Naphthalene	Phenanthrene	Anthracene	Pyrene
	mmol N-NH_4_ kg^−1^ DM soil h^−1^			
0	2.76 c^a^	2.76 d	2.76 d	2.76 d
1000	2.00 b	2.43 c	1.93 c	2.22 c
2000	1.80 b	1.44 b	1.57 b	1.63 b
4000	1.11 a	1.06 a	1.18 a	1.14 a
average	1.91	1.92	1.86	1.93
*r*	−0.97	−0.95	−0.93	−0.98

^a^In columns homogenous groups are followed by the same letter

*r* correlation coefficient

### Soil resistance (RS) to contamination with PAHs

There is a general scarcity of studies investigating soil resistance based on the activity of soil enzymes (Wyszkowska et al. 2013; Lipińska et al. 2014a, b). Soil resistance is generally defined as the stability of the soil ecosystem after exposure to external factors, mostly anthropogenic stressors. Other factors include soil type, type of land use, and climate (Orwin and Wardle 2004). Orwin and Wardle (2004) proposed a method for calculating RS values in the range of −1 to 1. RS of 1 implies 100 % soil resistance and an absence of environmental stressors. The closer the RS values are to −1, the greater the influence of external factors, and RS of −1 points to 200 % influence of the stressor. The resistance of loamy sand, calculated based on dehydrogenase and urease activity, varied subject to the dose and type of PAHs. RS values decreased with an increase in soil contamination with naphthalene, phenanthrene, anthracene, and pyrene (Table 5). The lowest average RS values were noted in soil treated with phenanthrene. The highest average RS values were observed in soil contaminated with anthracene, which was characterized by the smallest drop in dehydrogenase activity and in soil treated with pyrene, which was characterized by the lowest decrease in urease activity.

**Table 5:** Soil resistance (RS) to contamination with PAHs

Dose of PAHs (mg kg^−1^ DM soil)	Type of PAHs			
	naphthalene	phenanthrene	anthracene	pyrene
Dehydrogenases				
1000	0.929 c^a^	0.700 c	0.956 b	0.981 c
2000	0.847 b	0.641 b	0.942 b	0.859 b
4000	0.687 a	0.427 a	0.839 a	0.709 a
average	0.821	0.589	0.912	0.849
r	−0.99	−0.99	−0.97	−0.99
Urease				
1000	0.571 c	0.164 b	0.539 c	0.672 c
2000	0.483 b	0.091 a	0.399 b	0.419 b
4000	0.254 a	0.065 a	0.274 a	0.260 a
average	0.436	0.106	0.404	0.450
r	−0.99	−0.89	−0.97	−0.94

^a^In columns for individual enzymes homogenous groups are followed by the same letter

*r* correlation coefficient

### Bacteria occurring in a soil contaminated with PAHs

Anthracene and pyrene had the most stimulating influence on the abundance of organotrophic bacteria and soil-dwelling microorganisms. The highest total counts of bacteria and molds were determined in soil samples treated with anthracene, where microbial abundance was 1.34-fold and 4-fold higher than in control, respectively, (Table 7). Staphylococci proliferated most rapidly in soil contaminated with pyrene where microbial counts increased 2.16-fold in comparison with control. The highest growth rate of *Enterobacteriaceae* on VRBG agar was reported in samples treated with naphthalene (355.5-fold higher in comparison with control) (Table 6). Anthracene, a three-ring PAH, is often used in studies of PAH biodegradation. During 32 weeks of incubation, microorganisms exposed to high doses of anthracene gradually decomposed and utilized hydrocarbons as a source of carbon in the process of adapting to new environmental conditions. The second most influential PAH was pyrene, a model compound in studies investigating the biodegradation of high-molecular-weight hydrocarbons. High pyrene doses probably contributed to the gradual release of carbon, which increased staphylococci counts.

**Table 6:** The number of bacteria, staphylococci, molds, *Enterobacteriaceae* in soil contaminated with PAHs

Microorganisms	Control	Naphthalene	Phenanthrene	Anthracene	Pyrene
Number of microorganisms (10^7^ cfu kg^−1^ DM soil)					
Number of bacteria	115 ± 18	38 ± 4	90 ± 9	154 ± 14	43 ± 7
Number of microorganisms (10^3^ cfu kg^−1^ DM soil)					
Staphylococci	240 ± 22	430 ± 25	250 ± 15	450 ± 21	520 ± 32
Molds	9 ± 2	9 ± 1	8 ± 2	36 ± 7	25 ± 4
*Enterobacteriaceae*	9 ± 3	3200 ± 103	300 ± 21	1600 ± 86	200 ± 20

Bacterial isolates were identified to the species level by 16S-rDNA sequence analysis because every taxon has one or more unique 16S-rDNA nucleotide sequences. Nucleotide sequences of every tested isolate with the length of 1400 bp were amplified and sequenced, and PCR efficiency was validated by electrophoresis. The majority of bacteria cultured on PCA belonged to the genus *Bacillus* (Table 7), which is widely used in PAH degradation. Based on an analysis of the NCBI database, the greatest sequence similarity of bacteria cultured on PCA in the control sample was noted for *Bacillus frigoritolerans*, *Bacillus simplex*, and unclassified strains of the genus *Bacillus* with 99 % sequence similarity. The species identified in soil samples containing naphthalene were *B. frigoritolerans*, *Bacillus thuringiensis*, *Bacillus muralis*, *B. simplex*, and unclassified strains of the genus *Bacillus*. The strains isolated from phenanthrene-contaminated soil were identified as *B. frigoritolerans*, *B. muralis*, *B. simplex*, and unclassified strains of the genus *Bacillus*. The following taxa were identified in soil contaminated with anthracene: *Bacillus pumilus*, *Bacillus safensis*, and unclassified strains of the genus *Bacillus*. The soil sample treated with pyrene was colonized by bacterial species of *B. frigoritolerans*, *B. thuringiensis*, *B. simplex*, and unclassified strains of the genus *Bacillus*.

**Table 7:** Microbial strains identified in soil samples contaminated with PAHs

Type/species	Type of PAHs				
	Control	Naphthalene	Phenanthrene	Anthracene	Pyrene
Strains from PCA medium					
*Bacillus frigoritolerans*	+	+	+	−	+
*Bacillus simplex*	+	+	+	−	+
*Bacillus thuringiensis*	−	+	−	−	+
*Bacillus muralis*	−	+	+	−	−
*Bacillus pumilus*	−	−	−	+	−
*Bacillus safensis*	−	−	−	+	−
Unclassified strains of *Bacillus*	+	+	+	+	+
Strains from Baird-Parker agar					
*Bacillus psychrodurans*	+	−	−	−	−
*Bacillus pumilus*	−	−	+	−	+
*Bacillus safensis*	−	−	+	−	+
*Bacillus aerophilus*	−	−	+	−	+
*Bacillus altitudinis*	−	−	+	−	−
*Corynebacterium amycolatum*	−	−	−	+	−
*Paenibacillus alvei*	−	+	−	−	−
*Paenibacillus apiarius*	−	+	−	−	−
*Paenibacillus taiwanensis*	−	+	−	−	−
Unclassified strains of *Bacillus*	+	−	+	−	+
Unclassified strains of *Paenibacillus*	−	+	−	−	−
Unclassified strains of *Corynebacterium*	−	−	−	+	−

“+” presence of the strain in soil sample; “−” absent in soil sample

**Fig. 4:**
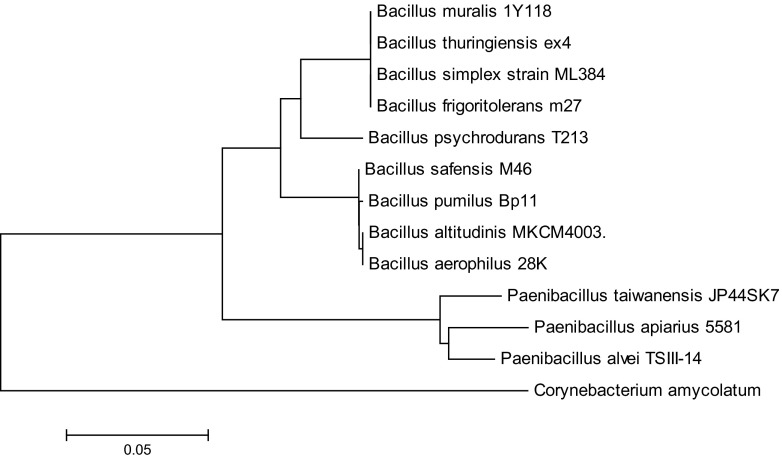
Phylogenetic tree of genetic similarity between selected bacterial strains isolated from PAH-contaminated soil

The following Staphylococcus species were identified on Baird-Parker agar in the control sample: *Bacillus psychrodurans* and unclassified strains of the genus *Bacillus*. Based on an analysis of the NCBI database, the greatest sequence similarity was noted for *Paenibacillus alvei*, *Paenibacillus apiarius*, *Paenibacillus taiwanensis*, and unclassified strains of the genus *Paenibacillus*. Soil contaminated with phenanthrene was colonized by *B. pumilus*, *B. safensis*, *Bacillus aerophilus*, *Bacillus altitudinis*, and unclassified strains of the genus *Bacillus*. The bacterial species identified in samples treated with anthracene were *Corynebacterium amycolatum* and unclassified strains of the genus *Corynebacterium*, and in samples contaminated with pyrene—*B. pumilus*, *B. safensis*, *B. aerophilus*, and unclassified strains of the genus *Bacillus*. The phylogenetic tree was developed to illustrate evolutionary correlations between the most characteristic strains isolated from PAH-contaminated soil (Fig. 4).

The majority of bacteria identified in this study belonged to the genus *Bacillus* sp., and their significance had been described in earlier research. The potential role of *Bacillus* sp. species in degrading PAHs was explored in numerous reports (Toledo et al. 2006; Seo et al. 2009). Pelaez et al. (2013) observed a reduction in PAH contamination levels after the application of autochthonous bacteria of the genera *Bacillus* and *Pseudomonas*. Moscoso et al. (2012) examined the biodegradation of three PAHs: phenanthrene, pyrene, and benzo(a)anthracene. A consortium of *Staphylococcus warneri* and *B. pumilus* effectively reduced 85 % of each of the tested hydrocarbons in a laboratory and degraded more than 90 % of the analyzed PAHs in a bioreactor over a period of three days. *Bacillus fusiformis* isolated from wastewater sludge contaminated with petroleum can be used to degrade naphthalene. Under optimal conditions, i.e., at a temperature of 30 °C and pH of 7, naphthalene was removed by the analyzed strain in 99.1 % in four days (Lin et al. 2010). Das and Mukherjee (2007) studied the effectiveness of the *Bacillus subtilis* DM-04 strain and reported a significant reduction in the levels of petroleum- and oil-derived PAHs. In the work of Xiao et al. (2012), a biosurfactant produced by *B. subtilis* stimulated mycelial growth under exposure to PAHs. Inoculation with the biosurfactant produced by *B. subtilis* and *Glomus etunicatum* fungi significantly reduced phenanthrene concentrations in soil and increased the activity of catalase and polyphenol oxidase.

### Effect of PAHs on seed germination and root growth of: *Lepidium sativum*, *Sorghum saccharatum*, and *Sinapis alba*

The effects of PAHs were determined based on analysis of seed germination and root growth in cress, sweet sorghum, and white mustard. Regardless of the applied PAH dose, seed germination was most inhibited in *L. sativum* (11.6 %), followed by *S. alba* (6.6 %) and *S. saccharatum* (1.6 %). A decrease in root length relative to control was observed in *L. sativum* and *S. alba* where root growth was inhibited by 17.7 and 28.0 % on average, respectively, (Table 8). The most PAH-resistant plant was sweet sorghum whose roots increased in length under exposure to PAHs in comparison with control. Irrespective of the plant species, seed germination was most inhibited by pyrene (11.1 %), whereas phenanthrene had the most inhibitory effect on root growth (20.6 %) in the analyzed plants.

**Table 8:** Effects of PAHs on seed germination and root growth of *Lepidium sativum*, *Sorghum saccharatum*, and *Sinapis alba*

Plant	Type of PAHs			
	Naphthalene	Phenanthrene	Anthracene	Pyrene
Inhibition of seed germination (%)				
*L. sativum*	20.0 a^a^	10.0 abc	0.0 c	16.6 ab
*S. saccharatum*	0.0 c	0.0 c	6.6 abc	0.0 c
*S. alba*	6.6 abc	0.0 c	3.3 bc	16.6 ab
Inhibition of root growth (%)				
*L. sativum*	16.2 cd	22.5 abc	16.4 cd	15.9 cd
*S. saccharatum*	9.3 cd	−1.25 d	−22.7 e	−23.2 e
*S. alba*	21.8 bc	40.4 a	37.9 ab	11.9 cd

Negative values indicate PAHs’ stimulating effect on root growth in the examined plant species

^a^Homogenous groups for inhibition of seed germination and root growth are followed by the same letter

Root growth was inhibited or stimulated subject to plant species and the applied PAHs. The greatest root elongation was reported in *S. saccharatum*, which was most resistant to PAH contamination. A stimulating influence of PAHs on plant growth was also reported by other authors (Smreczak and Maliszewska-Kordybach 2003). When applied at the dose of 4000 mg kg^−1^ soil DM, each of the analyzed PAHs were toxic for *L. sativum* and *S. alba*. Smreczak and Maliszewska-Kordybach (2003) demonstrated that a mixture of PAHs inhibited plant growth already at the dose of 100 mg kg^−1^ soil DM. In our studies, phenanthrene had the most inhibitory effect on the growth of all evaluated plants. Phenanthrene was the hydrocarbon which also inhibited the development of organotrophic bacteria and the activity of dehydrogenases to the greatest extent. The count of bacteria in the root zone of the tested plants, reduced under the influence of phenanthrene, could be the reason for the inhibition of the root growth. An opposite trend was recorded following the application of pyrene to the soil, which stimulated the count of microorganisms, while regardless of the plants being tested, the hydrocarbon inhibited the growth of their roots to the smallest extent. This could be related to both the progressive degradation of pyrene in the soil and the adaptation of plants to adverse conditions through an increase in the secretion of oxidoreductases released by the underground parts. Under the influence of polycyclic aromatic hydrocarbons, an increase in the microorganism population in the root zone of *L. sativum*, *S. alba*, and *S. saccharatum* could occur, which, in combination with extracellular enzymes, resulted in the stimulation of the root growth (Muratova et al. 2009). The bioavailability of phenanthrene to *L. sativum* was determined by Bogolte et al. (2007) who observed strong correlations between PAH accumulation in plants and extractability under mild conditions.

## Conclusions

It was found that the tested PAHs increased the average colony counts of organotrophic soil bacteria; pyrene did so to the greatest extent (2.2-fold relative to non-contaminated soil), phenanthrene to the smallest extent (1.4-fold relative to non-contaminated soil). None of the PAHs changed the value of the bacterial colony development (CD) index, while anthracene and pyrene increased the value of the eco-physiological (EP) diversity indicator. PAHs lowered the activity of the tested enzymes. The activity of dehydrogenases was diversified to a greater extent by the type of hydrocarbon (54.56 %) rather than by the dose (10.64 %) thereof, while for the activity of urease, the situation was the opposite: greater extent by dose (95.42 %) than by type (0.21 %). Dehydrogenases are characterised by greater resistance to the action of PAHs than urease. Based on seed germination and root growth, it has shown that *S. alba* is best suited, being the most vulnerable plant, while *S. saccharatum* is the least suited. Subjecting a soil to strong pressure of PAHs leads to disturbances to the biological parameters of the soil, seed germination, and root growth *L. sativum*, *S. saccharatum*, and *S. alba*.
